# Supplementary material for: Surface Microbiome Profiling of Dental Elevators Using Third‐Generation Sequencing: Implications for Infection Control in Dental Practice
Source: Microbiologyopen. 2025 Dec 8;14(6):e70178. doi: 10.1002/mbo3.70178 (PMC12685768; doi:10.1002/mbo3.70178)
Supplement: Supplementary file 1 — customer_backup. [file MBO3-14-e70178-s001.zip › customer_backup/customer_backup/function_analysis/BugBase/treat/otu_contributions/genus/taxa_legend.pdf]

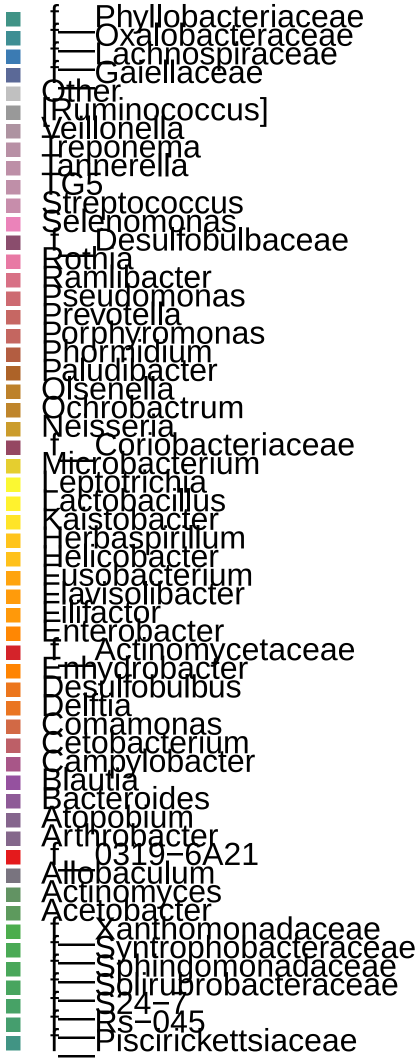
f__Phyllobacteriaceae
f__Oxalobacteraceae
f__Lachnospiraceae
f__Gaiellaceae
Other
[Ruminococcus]
Veillonella
Treponema
Tannerella
TG5
Streptococcus
Selenomonas
f__Desulfobulbaceae
Rothia
Ramlibacter
Pseudomonas
Prevotella
Porphyromonas
Phormidium
Paludibacter
Olsenella
Ochrobactrum
Neisseria
f__Coriobacteriaceae
Microbacterium
Leptotrichia
Lactobacillus
Kaistobacter
Herbaspirillum
Helicobacter
Fusobacterium
Flavisolibacter
Filifactor
Enterobacter
f__Actinomycetaceae
Enhydrobacter
Desulfobulbus
Delftia
Comamonas
Cetobacterium
Campylobacter
Blautia
Bacteroides
Atopobium
Arthrobacter
f__0319−6A21
Allobaculum
Actinomyces
Acetobacter
f__Xanthomonadaceae
f__Syntrophobacteraceae
f__Sphingomonadaceae
f__Solirubrobacteraceae
f__S24−7
f__Rs−045
f__Piscirickettsiaceae
